# Text mining for disease surveillance in veterinary clinical data: part one, the language of veterinary clinical records and searching for words

**DOI:** 10.3389/fvets.2024.1352239

**Published:** 2024-01-23

**Authors:** Heather Davies, Goran Nenadic, Ghada Alfattni, Mercedes Arguello Casteleiro, Noura Al Moubayed, Sean O. Farrell, Alan D. Radford, Peter-John M. Noble

**Affiliations:** ^1^Institute of Infection, Veterinary and Ecological Sciences, University of Liverpool, Liverpool, United Kingdom; ^2^Department of Computer Science, University of Manchester, Manchester, United Kingdom; ^3^Department of Computer Science, Jamoum University College, Umm Al-Qura University, Makkah, Saudi Arabia; ^4^Department of Computer Science, Durham University, Durham, United Kingdom

**Keywords:** big data, text mining, machine learning, neural language modeling, clinical records, companion animals

## Abstract

The development of natural language processing techniques for deriving useful information from unstructured clinical narratives is a fast-paced and rapidly evolving area of machine learning research. Large volumes of veterinary clinical narratives now exist curated by projects such as the Small Animal Veterinary Surveillance Network (SAVSNET) and VetCompass, and the application of such techniques to these datasets is already (and will continue to) improve our understanding of disease and disease patterns within veterinary medicine. In part one of this two part article series, we discuss the importance of understanding the lexical structure of clinical records and discuss the use of basic tools for filtering records based on key words and more complex rule based pattern matching approaches. We discuss the strengths and weaknesses of these approaches highlighting the on-going potential value in using these “traditional” approaches but ultimately recognizing that these approaches constrain how effectively information retrieval can be automated. This sets the scene for the introduction of machine-learning methodologies and the plethora of opportunities for automation of information extraction these present which is discussed in part two of the series.

## 1 Introduction

The advent of computerized clinical recording in veterinary medicine has enabled collation of large volumes of clinical data with a growing number of initiatives now attempting to leverage their value. A substantial portion of these data are tabular such as patient signalment and prescribing information. Additionally, data is available in the form of free-text clinical narratives entered by clinicians, nurses and technicians in the course of looking after these patients. In order to extract information that would be informative for disease surveillance and research into animal health, it would be ideal if all clinical features were recorded using a standardized encoding which captured features described in clinical narratives (e.g., historical information, clinical examination findings, conclusions, and treatment plans) as standardized codes. However, systems to encourage or enforce standardized clinical coding tend to meet with resistance, non-compliance, and inaccuracies (1, 2). Given the large scale at which data can be collected, it is not feasible to manually read and annotate these corpora of text. As a consequence it has become desirable to extract information from clinical narratives using computerized techniques. Methodologies ranging from keyword searching through to advanced machine-learning techniques, often using artificial neural network models, have been deployed to this task (3, 4). With the advent of affordable highly parallelized computer hardware, these methods have become accessible to researchers even on modest budgets. In this mini-series we aim to summarize the different methods employed for veterinary text mining for companion animal data including (in the second part of the series) discussion of the state of the art technologies now being deployed for use in this field.

## 2 Text-mining veterinary clinical records

### 2.1 What are veterinary clinical records?

It is important to consider the structure of clinical texts prior to choosing a specific approach to text-mining. Answering such a question involves a task called corpus analysis. This is a mathematical approach used in linguistics, computer science, and other fields to analyse large collections of free-text data, or corpora, in order to extract meaningful insights and patterns. It typically involves searching and characterizing large quantities of text data. Through corpus analysis, scientists can identify patterns of language use, such as common words, phrases and grammatical structures. These patterns can then be used to draw conclusions about language use in specific contexts or to inform the development of natural language processing and machine learning technologies. Overall, corpus analysis is a powerful tool for understanding the complexities of language use and the ways in which it is shaped.

Corpus linguistics is perhaps best illustrated by considering an example analysis. Since its inception in 2008, the Small Animal Veterinary Surveillance Network (SAVSNET) has been collecting electronic health records (EHRs) from veterinary consultations in a sentinel network of participating practices representing ~10% of the practices in the UK (5). Each EHR comprises signalment information (breed, age sex, neutering status) as well as prescribed drugs and, importantly the free text recorded at the time of the consultation. SAVSNET currently holds over 10 million clinical free text narratives which represent a substantial resource for investigation of disease patterns in companion animals. A corpus analysis of a subset of these data can provide some key information regarding this dataset and illustrates many of the features likely to be present in free-text clinical records in similar initiatives. We evaluated a sample of 3,523,070 de-identified and non-empty free-text veterinary clinical records from 1,182,308 patients from 408 veterinary premises between 2014 and 2018 representing 40 unique species (583,952 males, 598,355 females).

A standard linguistic investigation into the lexical composition (words and word collocations—words that appear together more often than by chance) of veterinary free-text records was performed. We employed a comprehensive approach utilizing three corpus analysis tools: AntConc (6), LancsBox (7), and Sketch Engine (8). These tools provide a core set of functions for exploring and analyzing textual data, such as concordance analysis, collocation analysis, keyword analysis, and frequency analysis. These clinical records comprised 260,383,905 tokens (individual words and punctuation), 204,482,562 of which were words, of which 892,060 could be considered unique words, which further reduced to 774,314 unique words if all letters were converted to lower case (this may discard some meaning depending on word context). Records comprised 17,083,128 sentences albeit the frequent absence of meaningful punctuation typical of such EHRs makes this definition somewhat unreliable.

**Figure 1:**
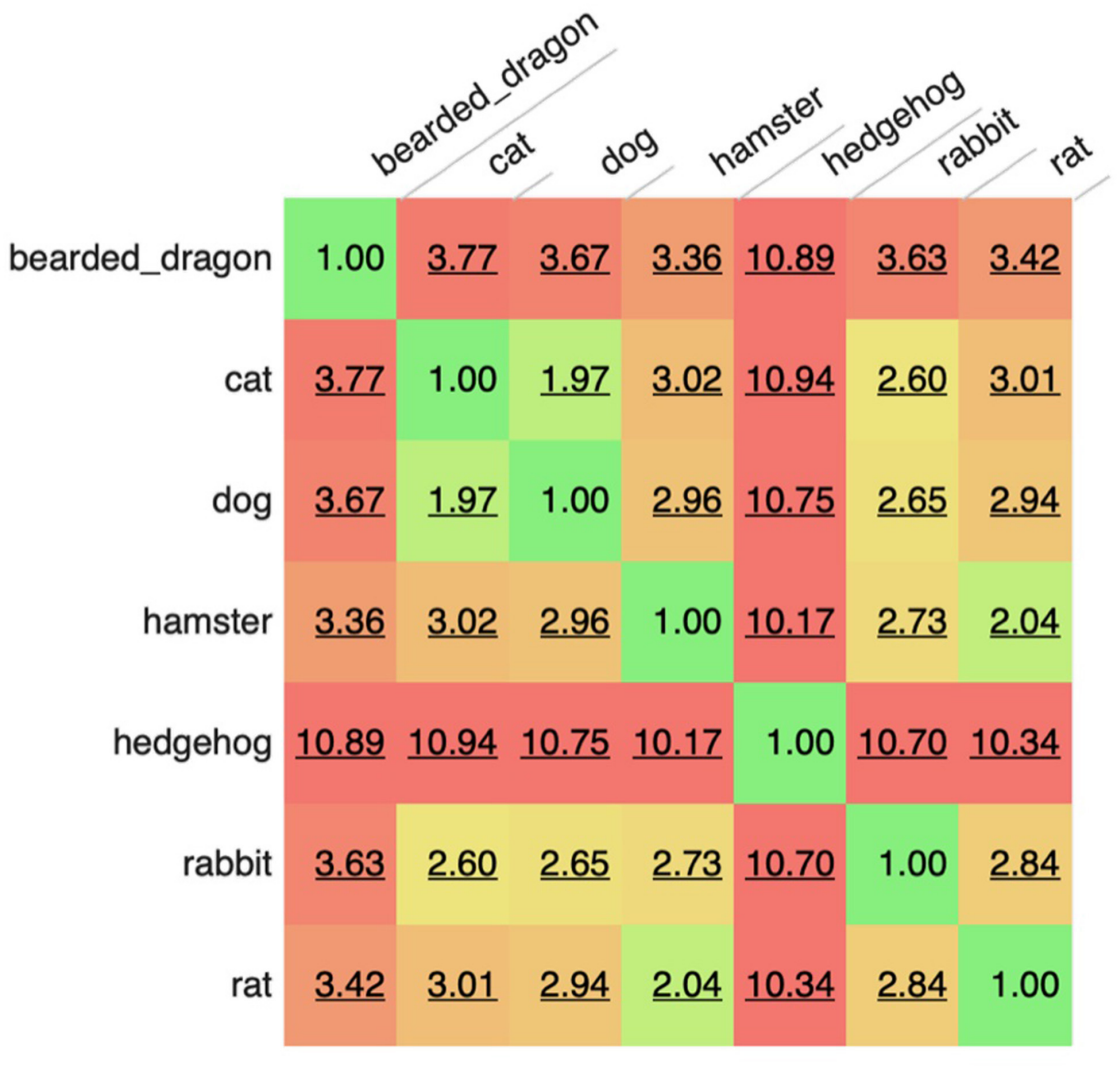
Comparison of sub-corpora of text based on species. A value of 1 indicates identical corpora. Higher scores reflect bigger differences between corpora. The score is not affected by sentence length, number of documents, corpus size or grammatical features, just the words/tokens present within each corpus.

The most common words were “owner” (1,086,418 occurrences), “normal” (984,730 occurrences), “well” (959,063 occurrences), “next” (836,754 occurrences), and “exam” (771,147 occurrences). The corpus included 340 words (and 56 bi- and tri-grams) that appeared more than 100,000 times in the corpus with 2,248 unique words accounting for 90% of all the words used and only 32,046 words accounting for 99% of all words used. However, there was an extended range of individual unique words (around 50 k out of 900 k) which appeared only once or twice in the corpus and in up to two documents (e.g., rppoduced, rppm4, hadpanacur, rpreast, and coppuple). These are likely a consequence of extensive misspellings present in such clinical free-text data. When manually reviewed for the clinical feature to which key-words referred using a modified CLEF nomenclature (9), the most common words belonged to test-results (referring to clinical findings) or healthcare activities (relating to varied aspects of case management). Analysis also revealed that frequent collocations or n-grams were most often concatenated tests-related words (e.g., “glucose\bilirubin\ketone”), findings or Healthcare activities relating to varied aspects of case management (e.g., “next appointment in”). When subsets of records (sub-corpora) were evaluated for similarity based on the specific words or n-grams in the each corpus, it could be seen that the most similar corpora were those for dogs and cats, followed by the rat and hamster. At the other extreme, some corpora like those for hedgehogs appeared to be very different. Such analyses suggest some high level commonality to how health records are constructed for related species and implies that careful attention may be needed when designing tools to evaluate these different corpora, such that a tool developed for one species may be less useful when applied in an more distantly related corpora (Figure 1; Table 1).

**Table 1:** Overview of sub-corpora metrics: token counts, unique tokens, records median lengths, and common *N*-grams.

**Subcorpus**	**Tokens**	**Unique tokens**	**Median record length**	**3–4 common *N*-grams**
dog	137,606,013	528,454	47	Next appointment in nothing abnormal detected pink and moist within normal limits mucous membranes pink
cat	52,893,928	271,826	45	Nothing abnormal detected next appointment in pink and moist within normal limits lab request references
rabbit	2,668,655	44,271	40	Next appointment in nothing abnormal detected within normal limits alert and responsive has no concerns
guinea_pig	1,070,161	27,269	52	Next appointment in nothing abnormal detected eating and drinking if no improvement alert and responsive
hamster	323,854	13,921	49	Next appointment in nothing abnormal detected put to sleep eating and drinking if no improvement
rat	301,048	13,450	47	Next appointment in nothing abnormal detected if no improvement eating and drinking alert and responsive
bearded_dragon	85,305	6,897	64	Next appointment in nothing abnormal detected Been in owners in owners possesion Been in owners possesion
hedgehog	307	212	29.5	—Nothing found—

Abbreviations and initialisms are common within EHRs with the most common being “O” (owner; 780,156 occurrences), NAD (no abnormalities detected; 477,903), abdo (abdomen; 379,912), BAR (bright alert and responsive; 349,470), palp (palpation; 283,319), HRn (heart rate normal; 256,256), OR (owner reports; 241,694), t (temperature; 234,501), DUDE (drinking urinating defaecating eating; 198,306), KC (kennel cough; 188,002), BCS (body condition score; 187,799), d (diarrhea; 173,757), ausc (auscultation; 150,863), CRT (capillary refill time; 106,482), and adv (advise; 92,803). Another study identified how some abbreviations were overloaded i.e., the same abbreviation meaning different things, for instance “rx” used to mean “treatment” in some narratives and “review” in others (10) and others displaying polysemy i.e., duplicated meaning in different forms (DUDE, EDUD, EDDU). An expanded list of over 400 abbreviations commonly encountered in veterinary EHRs together with their meaning is available through the SAVSNET website at http://www.liverpool.ac.uk/savsnet/publications/datasets.

The corpus analysis-derived answer to the question “What are veterinary clinical records”, shows that the clinical sub-language exemplified by the SAVSNET dataset is characterized by a high number of unique individual tokens many representing misspelling and abbreviations or initialisms and typical domain-specific word collocations all of which vary with the species being examined. Such linguistic studies are highly informative and foundational, highlighting the need to understand the common linguistic choices that will influence planning the automated processing of clinical narratives on a large scale. The importance of understanding the language of the corpus is perhaps best illustrated by those projects that rely on keyword searching to extract a subset of records from the entire corpus for further analysis; this approach of keyword searching is considered in the next section.

### 2.2 Keyword searching

As demonstrated by corpus analysis, the language used in veterinary free-text clinical narratives is extensive and diverse. These narratives can be interrogated using a variety of techniques in order to identify records of interest, the most basic of which is keyword searching. Despite the apparent simplicity of this approach, keyword searching can be a powerful tool for filtering large datasets. This is especially true in semi-structured data, where pre-defined words common to all documents can be used to identify the text of interest. For example, it would be reasonable to assume that a diagnosis would follow the text “Diagnosis:” or that a treatment plan would follow the text “Plan:”. Keyword searches have been very successfully deployed as an effective screen for candidate records for instance studying diabetes (11), hypoadrenocorticism (12), and patellar disease (13) with subsequent manual reading to filter out unwanted records. As an example of this approach, Heeley and colleagues screened for potential cases of diabetes by searching for the keywords diab, insul, hyperg, mell, glucose, DM, ketoa, ketou, IDDM, fruct, curve, insuv, prozi, canins, vetp, vet pen. This search illustrates the range of language used by veterinarians in EHRs, the need to use more than one keyword if many cases are not to be missed, and how truncation of keywords can reduce the number of terms required. For example the keyword diab will identify both diabetes and diabetic (11).

A pathology based-animal tumor registry was created using this keyword principle from laboratory data collected by the Small Animal Veterinary Surveillance Network (SAVSNET) (14). In this example, keyword matches were used to extract information relating to the tumor diagnosis and location from specific portions of what are essentially free text electronic pathology reports. The keywords “diagnosis” and either “prognosis” or “clinical history”, which are routinely present in such reports, were used as delimiters in order to extract the portion of text relating to the tumor diagnosis. Similarly, tumor location was extracted using the keywords “tumor diagnosis” or “clinical history” and “histology”. Keyword searches can also include rules, as demonstrated in this example where a prioritization system was used to search the three possible sections of the pathology report where the tumor location could be mentioned. Unfortunately such keyword delimiters are not so consistently present in EHRs from veterinary practice.

The utility of an “off the shelf” text-mining software for content analysis and keyword retrieval has also been investigated (3). Here, functions for identifying keywords and for the creation of more specific keyword lists were piloted. The results of this study highlight that this type of software could prove a valuable resource for practice-based research. However, the authors conclude that further work is required to validate this methodology in more complex populations where there is likely to be greater variation in the terminology used.

### 2.3 Rule-based searching

While keyword extraction is helpful for identifying mentions of the concept of interest in free-text, in many instances only a subset of these are relevant. In this situation, a rule-based approach can be taken to further guide the task.

Regular expressions, or regexes, were first patented in 1971. They consist of a sequence of characters defining rules for identifying a matching pattern within text (15) and are supported by many common programming languages including Python, Perl and JavaScript. Regexes can incorporate words, numbers, and non-alphanumeric characters and can therefore be used to develop complex patterns that express particular rules. We and others have successfully implemented this approach in a wide range of studies for example to identify mentions of grass seed foreign bodies (16), ear cropping (17), myxomatosis (18), ticks (19), fleas (20), and canine generalized epileptic seizures (21), amongst others. In these studies, regexes were developed to identify mentions of the concept of interest whilst disregarding instances where this concept was negated.

Many self help guides are available on the internet to help create regexes and need not be reproduced here but a simple exemplar can highlight the broad concepts. Here the following regex—(? < !b)(? < !inf)(? < !no)(? < !not)(lame | limp)—is used to identify either “lame” or “limp” as long as it is not preceded by a “b” (blame), “inf” (inflamed), “no” or “not” (negations). As before, this regex also uses keyword truncation—the search term lame will find lame and lameness just as limp will find limp and limping. Note “\s” is annotation for a space and other gap characters. The character “|” denotes OR. “? < !” denotes a negative look behind. Incorporating these negations in regexes is very important as it has been shown previously that up to 11% of sentences within veterinary free-text clinical narratives contain negated terms (22). Negation occurs to varying degrees such that in the corpus analysis above, negation of terms implying vomiting (i.e., “no/not vomiting,” “no v/d,” “no v+”) occurred 55 times more commonly than negation of the word seizure (i.e., “no seizures,” “no/not seizuring,” “no seizing”). It seems veterinary professionals are more likely to record the negation of certain clinical signs as part of their routine examination. Rule based systems for identification of negation have had some success but often work best in constrained document types for which quite specific rules can be implemented (23, 24). The above lameness regex has a relatively high positive predictive value (>90%), but false positives remain (e.g., no evidence of any limping) such that results of a regex still typically need to be read by domain experts to accept or decline them.

Because they follow rules, regexes also have the advantage of being highly predictable in their outcome. However, all but the most carefully crafted regex will still struggle to cope with the diversity of text typical of veterinary clinical records such that their results can still have low positive predictive values. And since most diseases are relatively rare in a given corpus, calculating good estimates for negative predictive value is also challenging. Studies using regexes frequently don't estimate the proportions of records missed by a regex that would still have met the case definition in question, and regex-based studies are therefore often not appropriate as a way of estimating the prevalence of a condition. As an example with the lameness regex above, it does not capture another term used by vets for lameness, namely “nwb” or non-weight bearing or inevitable spelling mistakes. Regular expressions have however proven particularly useful in compiling datasets for case control studies where retrieved records can be read by domain experts to identify those that meet a case definition for the study, whilst rejecting those that do not. One final point to consider when designing regexes (and keyword searches) is their potential to introduce bias. They are after all a selection process. This is again illustrated by our lameness regex above when we have missed consultations labeled as non-weight baring. In this example, perhaps by excluding the worst cases of lameness, we have biased our study to milder cases. Regular expressions are massively approachable by even relatively novice data scientists, and have proven invaluable in our early forays into text mining. However, some of their limitations mean they are now being superseded by neural language models (25).

Given the unstructured nature of veterinary clinical data, rules based methods can also be a useful tool for creating standardized data. This has proven particular invaluable when handling prescribing data, where mapping inconsistent product descriptions to specific veterinary products at scale can prove challenging. Previous SAVSNET research developed a text mining method to map practitioner-defined product descriptions to published taxonomies including the Veterinary Medicines Directorate (VMD) product database and the electronic Medicines Compendium (eMC) for human authorized products (26). In this work, an initial dataset of 52,267 product descriptions was manually reviewed against the two databases to determine those describing pharmaceutical agents. This dataset was then used to generate a list of prescription identifying strings for application to a larger dataset. Regular expressions were also utilized in order to exclude product descriptions which could be misclassified as veterinary products (for example, “phenobarbitone toxicity test”, which could otherwise be misclassified as a phenobarbitone prescription). This work allowed for analysis of large scale population level data relating to the treatments prescribed to companion animals in the UK (26). Such rule-based analysis can be built into very elaborate systems, for-instance, in combination with dictionaries of possible identifiers (personal-names and street names), SAVSNET is able to efficiently redact the majority of potential identifiers in records used for research (10).

The effectiveness of rule-based methods for classification tasks was also demonstrated in the large-scale extraction of antimicrobial treatment information from VetCompass Australia free-text clinical narratives (27). A rule-based algorithm was developed in order to map items dispensed in a consultation to a structured list of antimicrobials. A list of inventory items and a list of antimicrobials were tokenized and then an edited Levenshtein distance (i.e., the minimum number of operations required to transform one token into another) was calculated to measure the similarity between the tokens. For each token, this distance was divided by the length of the token and those with a ratio of <0.25 were considered to be a match. This algorithm identified antimicrobials with an accuracy of 96.7% and an F1 score of 0.85. The authors also tested several machine learning methods and it was found that the rule-based method was more efficient, highlighting the remaining value of such approaches.

Anholt et al. (28) used a rule-based approach for the identification of enteric syndrome (broadly synonymous with gastroenteric disease) in companion animal health records. Firstly, all unique words in the corpus were reviewed and those associated with enteric syndrome were used to create a categorization dictionary. This dictionary was used to scan a subset of the records to identify potential cases of enteric syndrome. From this subset, classification errors were identified including negated cases, general discussions about enteric syndrome (for example, the veterinary professional warning the owner to be aware of the potential for the syndrome to develop) and historical instances. Therefore, a new category was created in order to classify cases for exclusion. This category was based on a complex set of rules using Boolean operators and other modifiers occurring within a set distance of the mention of enteric syndrome. For example NO BEFORE #DIARRHEA/C 5 was used to exclude records where the word “no” appeared within five words of diarrhea, thereby excluding both “no diarrhea” as well as “no coughing, vomiting or diarrhea”. The resultant approach classified records with a sensitivity of 87.6% (95% CI 80.4–92.9%) and a specificity of 99.3% (95% CI, 98.9–99.6%).

## 3 Discussion

In this first part of the review series, we have covered the principles of corpus analytics, which can be used to characterize the challenge faced by text miners in terms of the make-up of veterinary language, informing interpretation of findings across the various sub-corpora that exist within these datasets (e.g., species-specific, time of day specific etc.). Armed with knowledge of this type, researchers can formulate plans for further analysis. Word co-location can help to decide on whether to use bigrams, trigrams or larger multi-word groupings in searching for specific key words or phrases. Keyword searches themselves work better where the text is constrained to short positive assertions (e.g., descriptive diagnostic field) or where concepts are seldom mentioned as a part of a negative phrase, for instance, while it is unusual to see “no seizures” as part of a routine clinical report, “no vomiting,” “no diarrhea,” or “no vdcs” are common phrases mandating careful evaluation if using simple keywords like “vomiting” and “diarrhea”.

In order to incorporate more context into word searching, rule-based systems provide more flexibility in defining the characteristics of text-sequences that define presence or absence of a specific feature. Whilst the creation of rules allows for flexible definition of the target text and can quite powerfully capture specific signals, rules can go out of date as new words enter the lexicon and different formats of reporting change how factors such as negation are articulated and thus may not be detected by rigid rules. As such signals based on word searching can degrade over time if not carefully maintained.

An understanding of the structure of the underlying corpus helps researchers design signal-detection strategies and is particularly valuable for development of keyword and rule-based text searching. Such methods have allowed some rapid progress in this “big data” era, and still have an important role to play in identifying subsets of records in a predictable and supervised manner. However, their strict rules means they are unlikely to ever fully cope with the complexity and grammatical errors present within many clinical records, such that the majority of meaning in even modest datasets of EHRs cannot be captured at suitable scales. In order to create more generalizable tools for feature detection and information extraction, machine learning is providing potent new avenues to annotate records at scale but a deep understanding of the corpus underlying these studies will aid researchers in understanding their outputs. Machine learning and AI approaches applicable to veterinary texts will be discussed in the second part of this review mini-series.

## Data availability statement

The raw data supporting the conclusions of this article will be made available by the authors, without undue reservation.

## Author contributions

HD: Writing—original draft, Writing—review & editing. GN: Formal analysis, Writing—original draft. GA: Formal analysis, Writing—original draft. MAC: Formal analysis, Writing—original draft. NAM: Writing—review & editing. SF: Writing—review & editing. AR: Writing—review & editing. P-JN: Writing—original draft, Writing—review & editing.
